# Methylation‐based alcohol consumption scores as prognostic biomarkers in colorectal cancer: Insights from a population‐based cohort

**DOI:** 10.1002/ijc.70086

**Published:** 2025-08-19

**Authors:** Tanwei Yuan, Katrin E. Tagscherer, Wilfried Roth, Melanie Bewerunge‐Hudler, Alexander Brobeil, Matthias Kloor, Hendrik Bläker, Hermann Brenner, Michael Hoffmeister

**Affiliations:** ^1^ Division of Clinical Epidemiology and Aging Research, German Cancer Research Center (DKFZ) Heidelberg Germany; ^2^ Medical Faculty Heidelberg, Heidelberg University Heidelberg Germany; ^3^ Institute of Pathology University Medical Center Mainz Mainz Germany; ^4^ Institute of Pathology Heidelberg University Hospital Heidelberg Germany; ^5^ Microarray Core Facility German Cancer Research Center (DKFZ) Heidelberg Germany; ^6^ Institute of Pathology University of Leipzig Medical Center Leipzig Germany; ^7^ German Cancer Consortium (DKTK), German Cancer Research Center (DKFZ) Heidelberg Germany

**Keywords:** alcohol consumption, colorectal cancer, DNA methylation, population‐based cohort, prognosis

## Abstract

Colorectal cancer (CRC) remains a leading cause of cancer‐related mortality, with alcohol consumption implicated in its etiology. However, alcohol's prognostic impact on CRC survival is unclear, and self‐reported intake is limited by bias. This population‐based cohort study evaluated blood DNA methylation‐based alcohol scores as objective prognostic tools in 2,129 CRC patients from Germany's DACHS study (2003–2021; median follow‐up: 10 years). Participants were recruited from 22 hospitals in Southwest Germany, including non‐metastatic (*n* = 1757) and metastatic (*n* = 372) patients with complete methylation and alcohol data. All three assessed methylation scores (3‐CpG, 450‐CpG, 144‐CpG) correlated with self‐reported lifetime/recent alcohol intake (Spearman's *r*: 0.29–0.36; *p* < 0.0001), particularly recent consumption. In non‐metastatic patients, self‐reported alcohol consumption showed a J‐shaped mortality risk, with elevated risks in heavy drinkers and abstainers. A similar dose–response pattern was observed for the 3‐CpG methylation score, which showed consistent and robust associations with increased overall mortality (adjusted hazard ratio [aHR] per standard deviation increase: 1.18, 95% CI: 1.11–1.25), non‐CRC‐related mortality (1.22, 1.13–1.32), and CRC‐specific mortality (1.12, 1.00–1.25). The 450‐CpG score was associated with overall mortality (1.07, 1.00–1.15), non‐CRC‐related mortality (1.14, 1.05–1.23), and alcohol consumption‐related mortality (1.59, 1.17–2.16). These findings highlight the potential utility of DNA methylation‐based alcohol scores, especially the 3‐CpG and the 450‐CpG scores, as prognostic tools for CRC outcomes. Such biomarkers may provide a more objective measure of alcohol exposure and complement self‐reported data in risk stratification and clinical decision‐making, though further validation is warranted before clinical implementation.

## What's New?

Alcohol's prognostic role in colorectal cancer remains uncertain, partly due to biases in self‐reported intake. This is the first study to evaluate blood DNA methylation‐based alcohol consumption scores as prognostic biomarkers in colorectal cancer. All three evaluated methylation scores were significantly correlated with self‐reported alcohol intake. The 3‐CpG score demonstrated robust and consistent associations with all‐cause and cause‐specific mortality in non‐metastatic patients. The 450‐CpG score was particularly associated with overall mortality, non‐CRC‐related mortality, and alcohol consumption‐related mortality. These findings highlight the potential of epigenetic biomarkers to provide objective exposure assessment and improve risk stratification in colorectal cancer care.

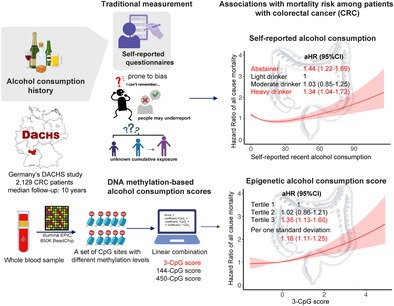

**Abbreviations**
aHR: adjusted hazard ratio
AUROC: area under the receiver operating characteristic curve
BMI: body mass index
CRC: colorectal cancer
IQR: interquartile range
MET: metabolic equivalent of task
NSAIDs: nonsteroidal anti‐inflammatory drug
STROBE: Strengthening the Reporting of Observational Studies in Epidemiology
TNM: tumor node, metastasis staging system

## 1 INTRODUCTION

Colorectal cancer (CRC) is one of the most prevalent cancers worldwide and remains a leading cause of cancer‐related mortality.^1^, ^2^ Extensive epidemiological studies and meta‐analyses have identified alcohol consumption as a strong risk factor for CRC incidence, with evidence supporting a dose‐dependent association.^3^, ^4^, ^5^ However, its impact on CRC prognosis remains uncertain. A 2014 meta‐analysis found higher CRC mortality among heavy drinkers, with a J‐shaped dose–response relationship between alcohol intake and CRC mortality.^6^ A subsequent meta‐analysis in 2019 found improved survival for light and moderate pre‐diagnostic alcohol consumption, while the association was not significant for heavy drinkers.^7^ In contrast, a recent study found no statistically significant associations between alcohol consumption and CRC recurrence.^8^

A key limitation of prior studies is the variability in the assessment and categorization of alcohol intake.^9^ Self‐reported alcohol screening questionnaires, commonly used in these studies, are prone to information bias and may fail to reflect cumulative exposure or individual variability.^10^ DNA methylation, an epigenetic modification that regulates gene expression, has been shown to be influenced by alcohol consumption.^11^, ^12^, ^13^ Recent advances have led to the development of DNA methylation‐based alcohol consumption scores, providing a more objective and biologically relevant measure to complement self‐reported data.^14^, ^15^, ^16^

Although these methylation‐based scores have been linked to CRC risk in prior research,^17^ their role in predicting CRC prognosis remains unclear. Understanding the association between alcohol‐induced epigenetic changes and CRC survival outcomes could offer valuable insights into risk stratification and patient management. This study aimed to examine the association between methylation‐based alcohol consumption scores and mortality among patients with CRC in a population‐based cohort from Germany, to assess the potential of these scores as prognostic tools for clinical application.

## 2 METHODS

### 2.1 Study cohort

This study adhered to the Strengthening the Reporting of Observational Studies in Epidemiology (STROBE) guidelines.^18^ Our analysis is based on data from the DACHS study, a large‐scale population‐based case–control and patient cohort study on CRC conducted in the Rhine‐Neckar region in southwest Germany between 2003 and 2021. The details of the DACHS study have been described previously.^19^ Participants included male and female German‐speaking patients over 30 years of age with a first‐time, histologically confirmed primary CRC diagnosis, who were physically and mentally capable of completing a 1‐h interview. Recruitment was conducted across 22 hospitals in the study region.

Only patients with complete data on lifetime alcohol consumption and DNA methylation were included in this analysis. Of the 5483 patients recruited from 2003 to 2016 with at least one follow‐up record, we excluded 28 patients without lifetime alcohol intake data. From the remaining 5455 patients, we finally included 2129 patients with blood methylation and 2270 with tumor tissue methylation data. These groups largely overlapped (1906 patients were included).

### 2.2 Data collection

Baseline data were collected shortly after CRC diagnosis through structured interviews conducted by trained interviewers. Information included sociodemographic factors, lifestyle habits, medical history, and disease symptoms. Tumor characteristics and disease stage (TNM 6th edition) were extracted from medical records.

Details of alcohol consumption assessment have been previously published. Briefly, alcohol consumption was assessed using self‐reported intake of beer, wine, and liquor at seven life stages (ages 20 to 80 years in 10‐year intervals) and for the 12 months prior to diagnosis. For each time point, average daily ethanol intake was estimated in grams per day for each beverage type using a food composition table^20^ (beer: 4 g/100 mL; wine: 8.6/100 mL; liquor: 33 g/100 mL). Mean daily intake was then used to calculate recent and lifetime alcohol consumption. Both recent and lifetime alcohol intake were categorized into sex‐specific groups as follows: 0, >0–12, >12–25, and >25 g/day for women, and 0, >0–24, >24–50, and >50 g/day for men, corresponding to abstainers, light, moderate, and heavy drinkers, respectively.

Follow‐up data were collected at 3, 5, and 10 years after diagnosis through standardized physician reports on therapy, comorbidities, and recurrence. Vital status, date, and cause of death were retrieved from population registries and public health authorities. The main causes of death were coded from the death certificates using the World Health Organization's guidelines based on the International Classification of Diseases, Tenth Revision (ICD‐10).^21^

### 2.3 DNA methylation‐based alcohol consumption score calculation

**FIGURE 1:**
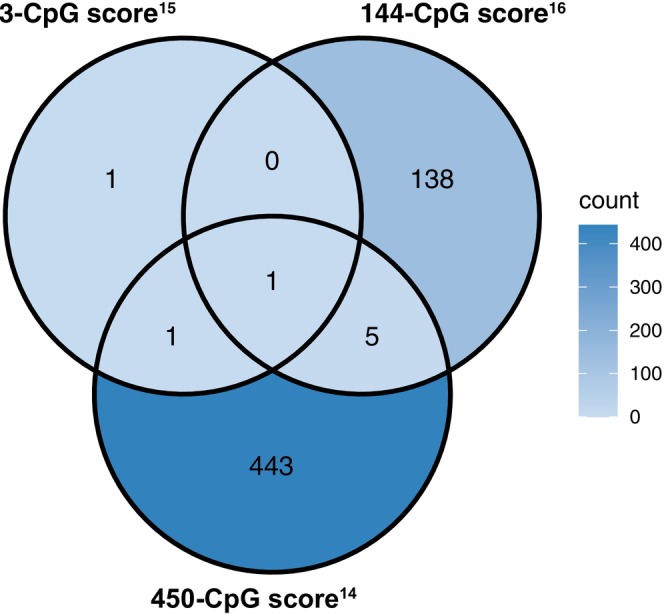
Overlapping CpGs of proposed blood methylation‐based alcohol consumption scores.

We used three blood‐based DNA methylation scores developed in prior studies to estimate alcohol consumption levels.^14^, ^15^, ^16^ Each score, based on CpG methylation, was calculated using linear equations provided by original studies.^14^, ^15^, ^16^, ^22^ The 3‐CpG score by Chamberlain et al.,^15^ 450‐CpG score by McCartney et al.,^14^ and 144‐CpG score by Liu et al.^16^ have all been externally validated in other cohorts.^17^, ^22^, ^23^ The majority of CpGs were unique to each score (Figure 1), except for cg06690548, which was shared among all three scores. To assess the performance of these scores across different sample types, we calculated them using methylation data from both blood and tumor tissues.

### 2.4 DNA methylation preprocessing

Single time‐point peripheral blood samples were collected a few weeks to a couple of months after CRC diagnosis and stored at −80°C. The median (interquartile range [IQR]) interval between diagnosis and blood collection was 36 (13–246) days. DNA methylation analysis was performed on blood samples from 2,129 patients with CRC diagnosed between 2003 and 2010 using the Infinium MethylationEPIC BeadChip Kit (Illumina, San Diego, CA, Research Resource Identifiers [RRID]: SCR_010233), covering over 850,000 CpG sites. Formalin‐fixed paraffin‐embedded tumor tissue sample blocks were immediately constructed after resection of the primary tumor and later collected from the pathological institutes serving the participating clinics. Details of DNA isolation from the tissue samples were described previously.^24^ DNA methylation analysis of tumor tissue from 2,270 patients diagnosed between 2003 and 2013 was performed using the Illumina Human Methylation 450 Bead‐Chip, covering over 485,000 CpG sites.

The same DNA methylation preprocessing procedures were applied to both tumor and blood samples. Raw DNA methylation data files generated from the iScan array scanner were processed using the “EpiSmokEr” R package,^25^ with ‘minfi’^26^ a core dependency. Missing CpG values were imputed using the nearest averaging of multiple imputations.^27^ Raw methylation data were normalized using the Illumina control probe normalization method, which scales intensities based on internal control probes provided on the Infinium BeadChip arrays. No additional background correction or dye‐bias adjustment was applied.^25^ We did not apply additional CpG filtering. Instead, CpG sites included in each score were directly extracted based on their availability in our dataset.

### 2.5 Statistical analysis

We did not perform a formal power analysis; instead, the sample size was determined based on available eligible patients in our cohort. Sociodemographic and clinical variables were summarized using descriptive statistics. Spearman correlation coefficients were used to assess associations between self‐reported alcohol consumption (lifetime and recent) and methylation‐based scores. Beverage‐specific analyses (beer, wine, liquor) were conducted, and score distributions across drinking categories were compared using box plots and the Jonckheere–Terpstra test. The discriminative power of methylation‐based scores for distinguishing heavy drinkers from non‐heavy drinkers was evaluated using the area under the receiver operating characteristic curve (AUROC) with 95% confidence intervals (2000 stratified bootstrap replicates). The concordance between methylation‐based alcohol scores measured in blood and tumor tissue was evaluated among participants with paired blood and tumor methylation data (*n* = 1906). Pearson correlation coefficients (r values) were calculated between each of the three methylation‐based scores across blood and tumor samples. Additionally, these correlations were stratified by sex, TNM stage, and self‐reported alcohol consumption categories.

Associations between alcohol exposure (self‐reported and methylation‐based) and all‐cause mortality were analyzed using Cox proportional hazards models. The proportional hazards assumption was verified using scaled Schoenfeld residuals, and the median follow‐up time was estimated using the reverse Kaplan–Meier method. Delayed‐entry Cox models accounted for the interval between CRC diagnosis and sample collection. Cause‐specific Cox proportional hazards models were used to analyze CRC‐ and non‐CRC‐related mortality, treating death from other causes as a competing risk. A sensitivity analysis was conducted using a cause‐specific Cox proportional hazards model to assess associations with deaths potentially due to alcohol consumption, including alcoholic liver disease, biliary cirrhosis, hepatocellular carcinoma, and unspecified liver cancer.

Cox models were adjusted for multiple covariables, including age, sex, tumor stage, tumor location, BMI at diagnosis, physical activity, smoking status, regular statin use, nonsteroidal anti‐inflammatory drug use, hormone replacement therapy, history of cardiovascular diseases, high blood pressure, prior large bowel endoscopy, and treatment (chemotherapy or radiotherapy). Missing covariate data were imputed 20 times using multiple imputation, with results combined using Rubin's rule.^28^ Sensitivity analyses were performed on complete cases.

Given that self‐reported abstainers are usually quite heterogeneous, we used light drinkers as the reference group.^9^ Methylation‐based scores were standardized, with one unit increase representing one standard deviation. Scores were also categorized into tertiles, with the lowest tertile serving as the reference. Dose–response analyses were conducted using restricted cubic splines to evaluate associations between self‐reported and methylation‐based alcohol exposure and mortality risk.

The primary survival analysis focused on stage I‐III CRC patients, with stage IV CRC patients analyzed separately. We also performed sensitivity analysis restricted to stage I‐III patients who did not receive chemotherapy or radiotherapy. Subgroup analyses were conducted according to sex for methylation‐based scores. Statistical significance was set at *p* value <0.05 in two‐sided testing. All statistical analyses were conducted in R version 4.2.0.

## 3 RESULTS

### 3.1 Characteristics of the study cohort

Patient characteristics were similar between the groups with blood methylation data and tumor tissue methylation data (Table 1), with comparable median ages (blood: 70; tumor: 69), sex distribution (blood: 41.1% female; tumor: 41.6% female), and percentage of stage IV CRC patients (blood, 13.7%; tumor, 14.2%). Most of the patients in both groups were lifetime light drinkers (approximately 56%); 17% were lifetime abstainers, and 7% were heavy drinkers. Recent heavy drinking was slightly more common (8%); 30% were recent abstainers.

**TABLE 1:** Characteristics of the analyzed study populations.

Characteristics	Blood sample (*N* = 2129)^a^	Missing	Tumor sample (*N* = 2270)^a^	Missing
Median age (IQR)	69.0 (62.0, 77.0)	0	70.0 (62.0, 77.0)	0
Female	875 (41.1)	0	945 (41.6)	0
Lifetime mean alcohol consumption behavior		0		0
Abstainers	366 (17.2)		389 (17.1)	
Light drinkers	1199 (56.3)		1270 (55.9)	
Moderate drinkers	403 (18.9)		440 (19.4)	
Heavy drinkers	161 (7.6)		171 (7.5)	
Recent mean alcohol consumption behavior		25 (1.2)		28 (1.2)
Abstainers	642 (30.2)		690 (30.4)	
Light drinkers	902 (42.4)		951 (41.9)	
Moderate drinkers	384 (18.0)		411 (18.1)	
Heavy drinkers	176 (8.3)		190 (8.4)	
TNM stage		12 (0.6)		6 (0.3)
I	379 (17.8)		411 (18.1)	
II	741 (34.8)		784 (34.5)	
III	705 (33.1)		746 (32.9)	
IV	292 (13.7)		323 (14.2)	
Chemotherapy or radiotherapy	1028 (48.3)	3 (0.1)	1061 (46.7)	6 (0.3)
Location		0		0
Colon	1363 (64.0)		1476 (65.0)	
Rectum	766 (36.0)		794 (35.0)	
BMI (kg/m^2^) at diagnosis		9 (0.4)		7 (0.3)
	46 (2.2)		49 (2.2)	
18.5–25	779 (36.6)		844 (37.2)	
25–30	891 (41.9)		953 (42.0)	
≥30	404 (19.0)		417 (18.4)	
Smoking status		2 (0.1)		3 (0.1)
Never smokers	982 (46.1)		1046 (46.1)	
Former smokers	820 (38.5)		882 (38.9)	
Current smokers	325 (15.3)		339 (14.9)	
Physical activity,^b^ Median (IQR)	216.0 (122.4)	34 (1.6)	210.9 (117.1)	27 (1.2)
Prior large bowel endoscopy	475 (22.3)	0	524 (23.1)	0
Use of hormone replacement therapy^c^	253 (29.0)	5 (0.5)	275 (29.1)	3 (0.3)
History of cardiovascular diseases	570 (26.8)	62 (2.9)	625 (27.5)	64 (2.8)
Use of statins	312 (14.7)	2 (0.1)	351 (15.5)	1 (0.0)
Use of NSAIDs	527 (24.8)	0	589 (25.9)	0
History of diabetes	397 (18.6)	14 (0.6)	425 (18.7)	12 (0.5)
History of high blood pressure	1098 (51.6)	26 (1.2)	1188 (52.3)	28 (1.2)

Abbreviations: BMI, body mass index; IQR, interquartile range; MET, metabolic equivalent of task; NSAIDS, nonsteroidal anti‐inflammatory drug; TNM, tumor, lymph node, and metastasis staging system.

^a^ The two sample groups contained 2008 overlapping patients.

^b^ Lifetime average MET‐hours/week.

^c^ Only among female participants.

The median follow‐up was 10.6 years for patients with analyzed blood samples (interquartile range [IQR]: 10.1–15.2) and 10.5 years for those with analyzed tumor tissue (IQR 10.1–12.5), with 1,175 (including 601 CRC‐related events) and 1,233 (including 639 CRC‐related events) deaths recorded in the blood and tumor tissue groups, respectively. The cumulative incidence curves of the two patient groups largely overlapped (Figure S1). The number of non–CRC‐related deaths was 550 in the blood sample cohort and 571 in the tumor sample cohort. Among these, the most frequent cause of death was unspecified heart failure (blood: *n* = 34; tumor: *n* = 38). Among patients with stage I–III CRC, the number of deaths potentially attributable to alcohol consumption was 13 in the blood sample cohort and 15 in the tumor sample cohort.

### 3.2 Associations between epigenetic and self‐reported alcohol consumption

**FIGURE 2:**
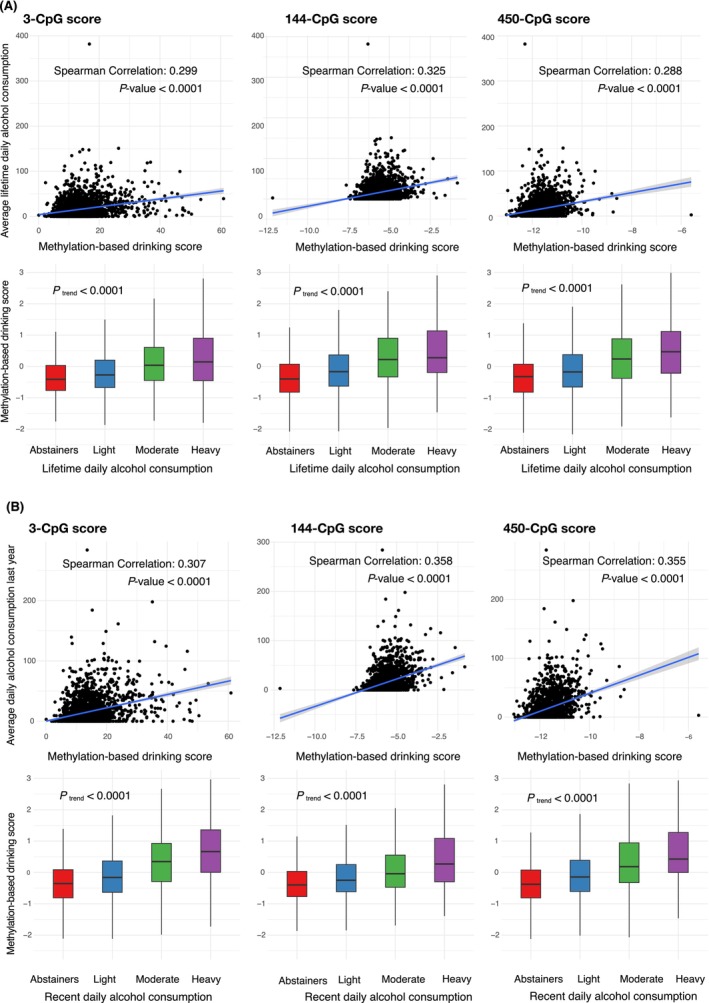
(A) Correlation between lifetime daily alcohol consumption and each of the three methylation‐based drinking scores (3‐CpG, 144‐CpG, 450‐CpG). (B) Correlation between recent daily alcohol consumption (past year) and methylation‐based drinking scores.

Among patients with blood methylation data, all three methylation‐based scores correlated significantly with self‐reported lifetime alcohol consumption (Spearman correlation coefficients, range: 0.29–0.33, *p* < 0.0001); they increased progressively across lifetime never, light, moderate, and heavy drinkers (*p*_trend_ <0.0001, Figure 2A).

Correlations were stronger for recent alcohol consumption (Spearman correlation coefficients, range: 0.31–0.36, *p* < 0.0001, Figure 2B). Beverage‐specific analyses (Figures S2 and S3) showed the strongest correlations with beer (median: 0.24), followed by wine (median: 0.22) and liquor (median: 0.11). All three scores showed good discriminative ability for recent heavy drinkers versus non‐heavy drinkers (median AUC: 0.73, Table S1), with score^14^ achieving the highest AUC (0.75 [0.71–0.78]). The discriminative ability was lower for lifetime consumption (median AUC, 0.67).

Using tumor methylation data, correlations with alcohol consumption were biologically irrelevant (Spearman correlation coefficients, range: 0.03–0.06); although trends in self‐reported consumption categories were statistically significant (Figure S4). Discriminative performance was poor, with AUC values below 0.60 (Table S1).

Correlations between tumor‐ and blood‐derived methylation scores were all very weak (Pearson *r* values, median: 0.09; range: 0.04–0.11; Figure S5). The correlation was slightly higher among self‐reported recent heavy drinkers (median r: 0.11), suggesting stronger external alcohol exposure signals in this subgroup. In contrast, compared with stage I‐III patients (median r: 0.07), correlations were notably lower among stage IV patients (median: 0.03).

### 3.3 Associations of self‐reported and methylation‐based alcohol consumption with mortality risk

**FIGURE 3:**
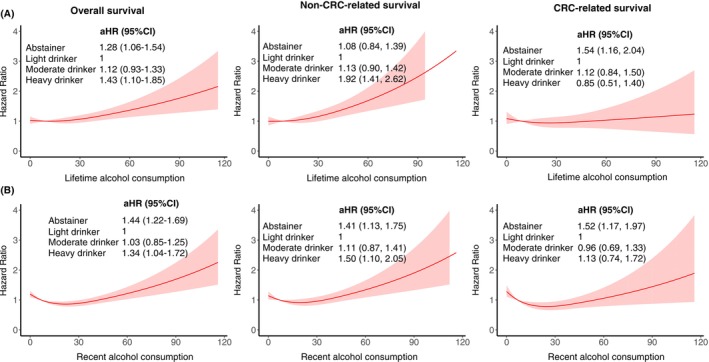
(A) Dose–response relationships between lifetime alcohol consumption and hazard ratios for overall survival, non‐CRC‐related survival, and CRC‐related survival. (B) Dose–response relationships between recent alcohol consumption (past year) and survival outcomes. The multivariate Cox model was adjusted for age, sex, tumor stage, tumor location, body mass index at diagnosis, physical activity, smoking status, regular statin use, nonsteroidal anti‐inflammatory drug use, hormone replacement therapy, history of cardiovascular disease, high blood pressure, prior large bowel endoscopy, and treatment with chemotherapy or radiotherapy. CI, confidence interval; CRC, colorectal cancer; HR, hazards ratio.

In patients with stage I‐III CRC, a J‐dose–response relationship was observed for self‐reported alcohol consumption and mortality (Figure 3). Both lifetime and recent heavy drinkers had increased overall mortality compared to light drinkers (lifetime: aHR: 1.43, 95%CI: 1.10–1.85; recent: 1.34, 1.04–1.72). Lifetime heavy drinking was also associated with higher non‐CRC‐related mortality (1.92, 1.41–2.62), with similar associations for recent heavy drinking (1.50, 1.10–2.05). However, heavy alcohol consumption was not significantly associated with CRC‐related mortality. Lifetime abstaining was associated with an increased overall risk (1.28, 1.06–1.54) and CRC‐related mortality (1.54, 1.16–2.04), whereas recent abstaining was associated with higher overall (1.44, 1.22–1.69), non‐CRC‐related (1.41, 1.13–1.75), and CRC‐related mortality (1.52, 1.17–1.97).

**FIGURE 4:**
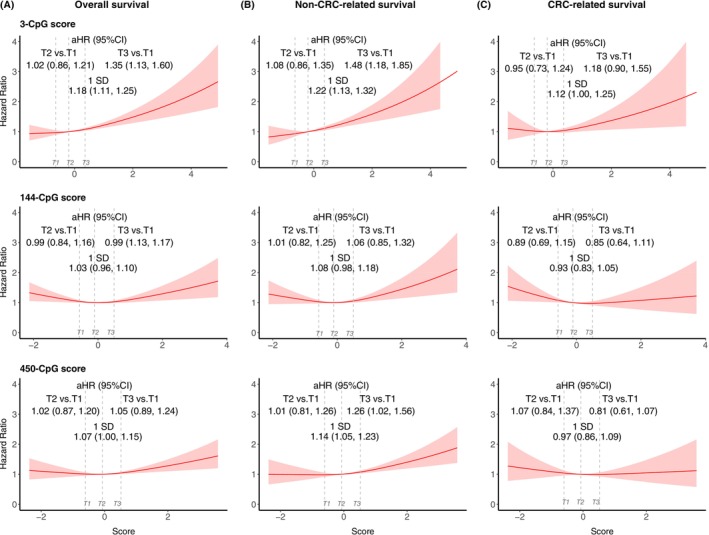
Associations between methylation‐based alcohol consumption scores and survival outcomes among patients with stage I‐III CRC. (A) Overall survival; (B) Non‐CRC‐related survival; and (C) CRC‐related survival in relation to three blood DNA methylation‐based alcohol scores. Red curves represent aHRs estimated using restricted cubic spline models; shaded areas indicate 95% CIs. Vertical dashed lines mark tertiles (T1–T3). aHRs and 95% CIs are shown for T2 vs. T1 and T3 vs. T1 comparisons as well as for a 1‐SD increase in score. The multivariate Cox model was adjusted for age, sex, tumor stage, tumor location, body mass index at diagnosis, physical activity, smoking status, regular statin use, nonsteroidal anti‐inflammatory drug use, hormone replacement therapy, history of cardiovascular disease, high blood pressure, prior large bowel endoscopy, and treatment with chemotherapy or radiotherapy. aHR, adjusted hazards ratio; CI, confidence interval; CRC, colorectal cancer; T, tertiles.

The 3‐CpG score^15^ showed strong and consistent associations with elevated risks of overall (1.18, 1.11–1.25), non‐CRC‐related (1.22, 1.13–1.32), and CRC‐related (1.12, 1.00–1.25) mortality (Figure 4). The dose–response curve for this score closely resembled that of self‐reported recent alcohol consumption. In contrast, the 144‐CpG score^16^ and 450‐CpG score^14^ exhibited a U‐shaped pattern with mortality outcomes. The 450‐CpG score^14^ was significantly associated with higher overall (1.07, 1.00–1.15) and non‐CRC‐related mortality (1.14, 1.05–1.23), but not with CRC‐related mortality. Importantly, only the 450‐CpG score showed a strong association with deaths potentially attributable to alcohol consumption (aHR: 1.59, 95% CI: 1.17–2.16, Table S2).

### 3.4 Sensitivity and subgroup analyses

The associations remained consistent when blood‐derived methylation scores were analyzed using tertiles and complete case analyses (Table S3). In the complete case analyses, the 144‐CpG score^16^ was significantly associated with non‐CRC‐related mortality (1.12, 1.02–1.24). However, when scores were derived from tumor tissue DNA methylation data (Table S3) or when analyses were restricted to stage IV CRC (Table S4), none of the methylation scores showed significant associations with mortality outcomes.

Among patients with stage I‐III CRC who did not undergo chemotherapy or radiotherapy (Table S5), the associations for blood‐derived methylation scores remained consistent. Notably, the 3‐CpG score^15^ derived from tumor tissue was statistically significantly associated with CRC‐related mortality (1.18, 1.02–1.36). In sex‐specific subgroup analyses (Table S6), the blood‐derived 3‐CpG score^15^ was consistently associated with poorer overall and non‐CRC‐related mortality in both males and females. However, the magnitude of these associations was slightly stronger in females than in males. In contrast, the 450‐CpG score^14^ showed significant associations with an elevated risk of overall and non‐CRC‐related mortality exclusively in male patients when derived from blood samples. However, when methylation scores were derived from tumor tissue samples, only the 450‐CpG score^14^ showed increased non‐CRC‐related mortality in female patients (1.16, 1.00–1.34).

## 4 DISCUSSION

In this large patient cohort study, we verified a strong correlation between three blood‐derived methylation‐based alcohol consumption scores^14^, ^15^, ^16^ and self‐reported alcohol intake. We further validated the association between self‐reported alcohol consumption and CRC mortality, emphasizing the J‐shaped relationship for mortality, increased risk in heavy drinkers, and reduced risk in light drinkers. Among the three methylation‐based alcohol consumption scores, the 3‐CpG score^15^ was consistently associated with overall, non‐CRC‐related, and CRC‐specific mortality among patients with non‐metastatic CRC. The 450‐CpG score^14^ was strongly associated with deaths attributable to alcohol‐related causes. However, the relevance of these blood methylation scores was markedly reduced when derived from tumor tissues.

Consistent with findings from other cohorts,^15^, ^17^, ^22^ we observed strong correlations between blood methylation‐based scores and self‐reported alcohol intake. Notably, the 3‐CpG score^15^ performed comparably well to more complex scores^14^, ^16^ despite its simplicity. Beverage‐specific analyses revealed the strongest correlations with beer and wine consumption, likely due to the high prevalence of these beverages in our study population. Interestingly, the scores correlated more strongly with recent alcohol consumption than with lifetime intake, consistent with a prior study suggesting that alcohol‐induced epigenetic changes may be partially reversible upon cessation.^29^ However, a longitudinal study has indicated that certain methylation changes persist after detoxification in patients with severe alcohol use disorder.^30^ The stronger associations with recent alcohol intake may also reflect reduced recall bias compared to lifetime consumption estimates.

Our findings align with those of previous research showing increased all‐cause and non‐CRC‐related mortality among heavy drinkers and heightened overall and CRC‐specific mortality among abstainers compared with light drinkers.^9^ The observed J‐shaped association may reflect a complex interplay of biological, behavioral, and social factors. From a biological perspective, heavy alcohol consumption promotes carcinogenesis and metastasis, potentially through mechanisms such as acetaldehyde accumulation^9^, ^31^ and *CCL5*‐induced autophagy.^32^ Conversely, light to moderate alcohol consumption, particularly wine, may exert beneficial effects via antioxidant and anti‐inflammatory pathways.^33^, ^34^ Compounds such as polyphenols, resveratrol, and anthocyanin in red wine have shown anti‐proliferative effects on CRC cells and synergy with chemotherapy.^34^, ^35^, ^36^, ^37^ However, the J‐shaped pattern may also be explained by reverse causality and confounding by socioeconomic status. Specifically, individuals with an otherwise increased mortality risk due to pre‐existing health conditions or poor general health may be advised to not drink alcohol at all.^7^ Additionally, those with lower socioeconomic status were more likely to experience alcohol‐related harm, which could bias observed associations.^38^

The blood derived 3‐CpG score^15^ showed the strongest and most consistent association with CRC prognosis, with dose–response curves similar to those for self‐reported recent alcohol consumption. Notably, when this score was derived from tumor tissue, it was the only one among the three methylation‐based scores to retain prognostic value for CRC‐specific mortality in stage I–III patients who had not received chemotherapy or radiotherapy. Among the three CpGs, one site (cg06690548) is located on the *SLC7A11* gene, which protects cancer cells from oxidative stress,^39^ and its high expression has been associated with poorer prognosis in various cancers, including CRC.^40^ Another site (cg00716257) resides on the *JDP2* gene, a tumor suppressor gene involved in tumor differentiation, apoptosis and immune regulation,^41^ with known relevance to CRC prognosis.^42^ These might explain why this score is the only score consistently and significantly associated with CRC‐related mortality.

The 450‐CpG score^14^ was associated with overall and non‐CRC‐related mortality but showed weaker associations compared to the 3‐CpG score.^15^ Notably, it was the only score strongly associated with deaths attributable to alcohol‐related causes. This finding suggests that the 450‐CpG score may be particularly sensitive to health impacts and epigenetic alterations specifically related to alcohol exposure. The 144‐CpG score^16^ exhibited even weaker prognostic associations despite stronger correlations with self‐reported alcohol intake, highlighting that self‐reported drinking may not fully reflect biologically relevant alcohol exposure. The lack of a protective association with lower methylation‐based scores, as observed in light drinkers, suggests that the epigenetic impact of light alcohol consumption may be subtle or confounded by other factors.

We observed sex‐specific differences in the prognostic relevance of methylation scores. The 3‐CpG score^16^ showed slightly stronger associations in females, whereas the 450‐CpG score^14^ was significant only in males. These differences may arise from metabolic and hormonal variations. Females typically exhibit higher blood alcohol levels and greater sensitivity to alcohol's toxic effects due to lower gastric alcohol dehydrogenase, smaller body size, and lower muscle‐to‐fat ratio.^43^ Conversely, alcohol's elevating effects on estrogen levels, which have anti‐inflammatory and antioxidant properties, could partially mediate the observed associations in females.^44^ The broader CpG coverage of the 450‐CpG score^14^ may capture epigenetic alterations that are influenced more by male‐specific factors.

When derived from tumor tissues, methylation scores were weakly correlated with self‐reported alcohol consumption and generally showed no significant association with CRC prognosis. While differences in the timing of blood and tumor sample collection may partly account for this discrepancy, it is more likely attributable to tumor‐specific factors, such as microenvironment changes and treatment effects,^45^, ^46^ which overshadow external exposure signals such as alcohol. This can be supported by our observation that the correlation between blood‐ and tumor‐based scores was lowest among patients with stage IV compared to those with stage I–III disease, suggesting a stronger influence of tumor‐induced epigenetic alterations in advanced cancer. These findings highlight the limited transferability of blood‐derived methylation scores to tumor tissues.

This study is among the first to extend the use of methylation‐based alcohol scores to prognosis in CRC patients using a large population‐based cohort with a long follow‐up period and high‐quality survival data. However, this study has several limitations that must be acknowledged. First, despite extensive adjustments for confounders, residual confounding from unmeasured variables such as socioeconomic status and detailed treatment regimens may remain. Second, the use of different methylation platforms for blood (EPIC BeadChip) and tissue (450 K BeadChip) samples could introduce variability, and the slightly different patient samples between analyses may have affected the comparability. Finally, the regional focus of the DACHS cohort on German‐speaking populations may have limited the generalizability of our findings.

This study underscores the practical value of blood methylation‐based alcohol scores as an objective tool for epidemiologists and clinicians to assess cumulative and recent alcohol consumption. These scores, particularly the 3‐CpG score^15^ and the 450‐CpG score,^14^ could complement the existing tools for patient stratification and guide clinical decision‐making in CRC management. Further research is required to validate these findings in diverse populations and cancer types. Moreover, future studies could investigate the integration of these scores with methylation‐based indicators of other lifestyle factors (e.g., smoking, body mass index)^14^, ^15^ and relevant clinical factors and explore the biological mechanisms underlying these associations.

## 5 CONCLUSIONS

In summary, this study highlights the potential utility of blood‐derived methylation‐based alcohol consumption scores as reliable biomarkers of alcohol exposure and prognostic indicators in CRC patients. Our findings offer valuable insights into risk stratification and personalized cancer management. Further validation and exploration of these scores across diverse populations in conjunction with other prognostic markers are warranted to enhance their clinical applicability and impact.

## AUTHOR CONTRIBUTIONS

**Tanwei Yuan:** Conceptualization; methodology; formal analysis; investigation; visualization; writing – original draft; writing – review and editing. **Katrin E. Tagscherer:** Data curation; writing – review and editing; project administration. **Wilfried Roth:** Data curation; writing – review and editing. **Melanie Bewerunge‐Hudler:** Writing – review and editing; data curation. **Alexander Brobeil:** Data curation; writing – review and editing. **Matthias Kloor:** Writing – review and editing; data curation. **Hendrik Bläker:** Data curation; writing – review and editing. **Hermann Brenner:** Conceptualization; supervision; funding acquisition; resources; writing – review and editing. **Michael Hoffmeister:** Conceptualization; project administration; funding acquisition; writing – review and editing; supervision.

## FUNDING INFORMATION

This study was supported by the German Research Council (BR 1704/6‐1, BR 1704/6‐3, BR 1704/6‐4, CH 117/1‐1, HO 5117/2‐1, HO 5117/2‐2, HE 5998/2‐1, HE 5998/2‐2, KL 2354/3‐1, KL 2354 3‐2, RO 2270/8‐1, RO 2270/8‐2, BR 1704/17‐1, BR 1704/17‐2), the Interdisciplinary Research Program of the National Center for Tumor Diseases (NCT), Germany, and the German Federal Ministry of Education and Research (01KH0404, 01ER0814, 01ER0815, 01ER1505A, 01ER1505B, and 01KD2104A).

## CONFLICT OF INTEREST STATEMENT

The authors declare no conflict of interest.

## ETHICS STATEMENT

The DACHS study was approved by the Ethics Committees of the Medical Faculty of Heidelberg University and the State Medical Boards of Baden‐Wuerttemberg and Rhineland‐Palatinate (approval number: 310/2001). All participants provided informed consent to participate in this study.

## Supporting information


**DATA S1.** Supporting information.

## Data Availability

The analysis code is available on https://github.com/TanweiY/methy_alcohol. Further information and the datasets used and analyzed during the current study are available from the corresponding author upon reasonable request.
