# Supplementary material for: Methylation‐based alcohol consumption scores as prognostic biomarkers in colorectal cancer: Insights from a population‐based cohort
Source: Int J Cancer. 2025 Aug 19;157(12):2521–31. doi: 10.1002/ijc.70086 (PMC12541559; doi:10.1002/ijc.70086)
Supplement: Supplementary file 1 — DATA S1. Supporting information. [file IJC-157-2521-s001.pdf]

Methylation-based alcohol consumption scores as prognostic biomarkers in colorectal cancer: insights from a population-based cohort

Tanwei Yuan, Katrin E. Tagscherer, Wilfried Roth, Melanie Bewerunge-Hudler, Alexander Brobeil, Matthias Kloor, Hendrik Bläker, Hermann Brenner, Michael Hoffmeister

Table of Contents

eTable 1. Discriminative power of DNA methylation-based scores by sample sources 2

eTable 2. Associations between methylation-based alcohol consumption scores and deaths potentially due to alcohol consumption[1] among stage I-III CRC patients..3

eTable 3. Associations between methylation-based alcohol consumption scores and mortality among patients with stage I-III CRC 4

eTable 4. Associations between methylation-based alcohol consumption scores and mortality among patients with stage IV CRC 6

eTable 5. Associations between methylation-based alcohol consumption scores and mortality among stage I-III patients did not receive chemotherapy or radiotherapy 7

eTable 6. Associations between methylation-based alcohol consumption scores and mortality by sex among patients with stage I-III CRC 8

eFigure 1 Cumulative mortality curves comparing patients with blood methylation data and those with tumor methylation data 9

eFigure 2 The associations between methylation-based alcohol consumption score and self-reported average lifetime daily beverage-specific alcohol intake 10

eFigure 3 The association between methylation-based alcohol consumption score and self-reported average recent daily beverage-specific alcohol intake 11

eFigure 4. Associations between tumor methylation-based alcohol consumption scores and self-reported daily alcohol consumption levels. A) Lifetime alcohol consumption; B) Recent alcohol consumption 12

eFigure 5. Pearson correlation r values between tumor- and blood-derived methylation scores among overlapping patients (*n* = 1906) overall, and by sex, TNM stage, and self-reported drinking behaviors. 13

eTable 1. Discriminative power of DNA methylation-based scores by sample sources

Score	AUC (95%CI)	
	**Blood sample**	**Tumor tissue**
**Lifetime heavy drinkers vs. non-heavy drinkers**		
3-CpG score	0.65 (0.60, 0.69)	0.55 (0.51, 0.60)
144-CpG score	0.67 (0.63, 0.71)	0.57 (0.52, 0.61)
450-CpG score	0.67 (0.63, 0.72)	0.58 (0.53, 0.62)
**Recent heavy drinkers vs. non-heavy drinkers**		
3-CpG score	0.69 (0.65, 0.74)	0.53 (0.49, 0.57)
144-CpG score	0.73 (0.69, 0.76)	0.53 (0.49, 0.57)
450-CpG score	0.75 (0.71, 0.78)	0.56 (0.51, 0.60)

AUC = area under the receiver operating characteristic curve; CI = confidence interval.

eTable 2. Associations between methylation-based alcohol consumption scores and deaths potentially due to alcohol consumption[1] among stage I-III CRC patients

	Blood sample	Tumor sample
**3-CpG score**		
Continuous	1.15 (0.70, 1.88)	0.79 (0.46, 1.36)
High vs. Low[2]	1.67 (0.48, 5.75)	0.75 (0.27, 2.14)
**144-CpG score**		
Continuous	1.24 (0.75, 2.06)	0.78 (0.45, 1.35)
High vs. Low	2.44 (0.65, 9.19)	0.49 (0.17, 1.46)
**450-CpG score**		
Continuous	1.59 (1.17, 2.16)	0.97 (0.61, 1.54)
High vs. Low	10.23 (1.32, 79.30)	0.61 (0.21, 1.74)

[1] Deaths potentially due to alcohol consumption, including alcoholic liver disease, biliary cirrhosis, hepatocellular carcinoma, and unspecified liver cancer.
[2] The cut-off was determined by median values.

eTable 3. Associations between methylation-based alcohol consumption scores and mortality among patients with stage I-III CRC

	aHR (95%CI)		aHR (95%CI)	
	Average over 20 imputed datasets[1]		Complete cases[2]	
	**Blood (N = 1918)**	**Tumor (N = 1946)**	**Blood (N = 1805)**	**Tumor (N = 1840)**
**Overall survival**				
3-CpG score				
Continuous	1.18 (1.11, 1.25)	1.03 (0.96, 1.10)	1.18 (1.10, 1.26)	1.03 (0.96, 1.10)
Tertile 2 v. Tertile 1	1.02 (0.86, 1.21)	1.05 (0.90, 1.24)	1.01 (0.84, 1.20)	1.05 (0.89, 1.24)
Tertile 3 v. Tertile 1	1.35 (1.13, 1.60)	1.07 (0.91, 1.26)	1.28 (1.07, 1.53)	1.06 (0.90, 1.26)
144-CpG score				
Continuous	1.03 (0.96, 1.10)	1.01 (0.94, 1.08)	1.06 (0.98, 1.14)	1.01 (0.94, 1.08)
Tertile 2 v. Tertile 1	0.99 (0.84, 1.16)	0.94 (0.80, 1.10)	0.98 (0.83, 1.16)	0.96 (0.81, 1.13)
Tertile 3 v. Tertile 1	0.99 (0.84, 1.17)	0.96 (0.82, 1.13)	1.02 (0.86, 1.21)	0.97 (0.82, 1.15)
450-CpG score				
Continuous	1.07 (1.00, 1.15)	0.99 (0.93, 1.05)	1.08 (1.00, 1.17)	0.99 (0.92, 1.06)
Tertile 2 v. Tertile 1	1.02 (0.87, 1.20)	1.00 (0.86, 1.18)	1.02 (0.86, 1.21)	1.01 (0.85, 1.19)
Tertile 3 v. Tertile 1	1.05 (0.89, 1.24)	1.07 (0.91, 1.25)	1.07 (0.90, 1.28)	1.06 (0.90, 1.25)
**Non-CRC-related survival**				
3-CpG score				
Continuous	1.22 (1.13, 1.32)	1.02 (0.93, 1.11)	1.23 (1.13, 1.34)	1.03 (0.94, 1.12)
Tertile 2 v. Tertile 1	1.08 (0.86, 1.35)	0.95 (0.77, 1.17)	1.07 (0.85, 1.36)	0.95 (0.77, 1.18)
Tertile 3 v. Tertile 1	1.48 (1.18, 1.85)	1.00 (0.81, 1.23)	1.45 (1.15, 1.84)	1.02 (0.82, 1.26)
144-CpG score				
Continuous	1.08 (0.98, 1.18)	0.99 (0.91, 1.08)	1.12 (1.02, 1.24)	0.97 (0.89, 1.06)
Tertile 2 v. Tertile 1	1.01 (0.82, 1.25)	0.98 (0.80, 1.20)	0.97 (0.77, 1.22)	0.97 (0.78, 1.19)
Tertile 3 v. Tertile 1	1.06 (0.85, 1.32)	0.92 (0.74, 1.13)	1.09 (0.87, 1.36)	0.87 (0.70, 1.09)
450-CpG score				
Continuous	1.14 (1.05, 1.23)	0.98 (0.90, 1.06)	1.17 (1.06, 1.29)	0.95 (0.88, 1.04)
Tertile 2 v. Tertile 1	1.01 (0.81, 1.26)	1.02 (0.84, 1.26)	1.01 (0.80, 1.27)	1.01 (0.82, 1.25)
Tertile 3 v. Tertile 1	1.26 (1.02, 1.56)	1.08 (0.88, 1.33)	1.29 (1.03, 1.61)	1.02 (0.82, 1.27)
**CRC-related survival**				
3-CpG score				
Continuous	1.12 (1.00, 1.25)	1.06 (0.95, 1.17)	1.10 (0.98, 1.24)	1.04 (0.93, 1.16)
Tertile 2 v. Tertile 1	0.95 (0.73, 1.24)	1.25 (0.96, 1.61)	0.94 (0.71, 1.24)	1.25 (0.96, 1.63)
Tertile 3 v. Tertile 1	1.18 (0.90, 1.55)	1.20 (0.92, 1.56)	1.09 (0.82, 1.46)	1.15 (0.87, 1.52)
144-CpG score				
Continuous	0.93 (0.83, 1.05)	1.04 (0.94, 1.16)	0.95 (0.83, 1.08)	1.08 (0.96, 1.20)
Tertile 2 v. Tertile 1	0.89 (0.69, 1.15)	0.88 (0.68, 1.14)	0.94 (0.72, 1.23)	0.97 (0.73, 1.27)
Tertile 3 v. Tertile 1	0.85 (0.64, 1.11)	1.02 (0.79, 1.32)	0.89 (0.67, 1.18)	1.14 (0.88, 1.50)
450-CpG score				

	aHR (95%CI)		aHR (95%CI)	
	Average over 20 imputed datasets[1]		Complete cases[2]	
	**Blood (N = 1918)**	**Tumor (N = 1946)**	**Blood (N = 1805)**	**Tumor (N = 1840)**
Continuous	0.97 (0.86, 1.09)	1.02 (0.92, 1.13)	0.97 (0.86, 1.10)	1.06 (0.96, 1.19)
Tertile 2 v. Tertile 1	1.07 (0.84, 1.37)	0.99 (0.76, 1.28)	1.08 (0.83, 1.40)	1.02 (0.77, 1.34)
Tertile 3 v. Tertile 1	0.81 (0.61, 1.07)	1.08 (0.84, 1.40)	0.83 (0.62, 1.10)	1.18 (0.90, 1.55)

CRC = colorectal cancer. aHR = adjusted hazards ratio; CI = confidence interval. The associations were evaluated by Cox regression models fully adjusted by age, sex, and tumor stage, tumor location, BMI at diagnosis, physical activity, smoking status, regular statin use, nonsteroidal anti-inflammatory drug use, hormone replacement therapy, history of cardiovascular diseases, high blood pressure, prior large bowel endoscopy, and treatment with chemotherapy or radiotherapy. [1]Missing covariable data were imputed 20 times using multiple imputation. Cox analyses were run across the 20 imputed datasets in parallel, with final results combined using Rubin's rule. [2] Missing covariable data were excluded, and only patients with complete information were included in the complete case analyses.

eTable 4. Associations between methylation-based alcohol consumption scores and mortality among patients with stage IV CRC

	aHR (95% CI)	
	**Blood sample (N = 292)**	**Tumor sample (N = 324)**
**Overall survival**		
3-CpG score	1.10 (0.93, 1.29)	0.96 (0.86, 1.07)
144-CpG score	0.91 (0.79, 1.05)	0.98 (0.88, 1.10)
450-CpG score	0.94 (0.80, 1.11)	0.92 (0.82, 1.04)
**Non-CRC-related survival**		
3-CpG score	0.71 (0.38, 1.32)	1.04 (0.58, 1.86)
144-CpG score	0.82 (0.40, 1.66)	0.94 (0.54, 1.64)
450-CpG score	0.95 (0.43, 2.08)	1.31 (0.81, 2.13)
**CRC-related survival**		
3-CpG score	1.12 (0.94, 1.32)	0.96 (0.86, 1.07)
144-CpG score	0.91 (0.78, 1.06)	0.98 (0.88, 1.10)
450-CpG score	0.94 (0.80, 1.11)	0.91 (0.80, 1.02)

aHR = adjusted hazards ratio; CI = confidence interv

eTable 5. Associations between methylation-based alcohol consumption scores and mortality among stage I-III patients did not receive chemotherapy or radiotherapy

	aHR (95% CI)	
	**Blood sample (N = 771)**	**Tumor sample (N = 783)**
**Overall survival**		
3-CpG score		
Continuous score	1.20 (1.07, 1.35)	1.05 (0.94, 1.16)
Tertile 2 vs. Tertile 1	1.02 (0.78, 1.32)	1.03 (0.79, 1.33)
Tertile 3 vs. Tertile 1	1.25 (0.96, 1.64)	1.12 (0.87, 1.46)
144-CpG score		
Continuous score	0.99 (0.88, 1.12)	1.03 (0.92, 1.14)
Tertile 2 vs. Tertile 1	0.97 (0.75, 1.25)	0.91 (0.71, 1.18)
Tertile 3 vs. Tertile 1	0.98 (0.75, 1.27)	1.06 (0.82, 1.37)
450-CpG score		
Continuous score	1.06 (0.94, 1.19)	0.97 (0.88, 1.07)
Tertile 2 vs. Tertile 1	1.14 (0.88, 1.47)	0.93 (0.73, 1.20)
Tertile 3 vs. Tertile 1	1.19 (0.92, 1.54)	0.97 (0.75, 1.25)
**Non-CRC-related survival**		
3-CpG score		
Continuous score	1.25 (1.05, 1.50)	0.90 (0.76, 1.06)
Tertile 2 vs. Tertile 1	0.96 (0.64, 1.45)	0.91 (0.63, 1.32)
Tertile 3 vs. Tertile 1	1.37 (0.92, 2.04)	0.84 (0.57, 1.25)
144-CpG score		
Continuous score	1.08 (0.90, 1.29)	0.98 (0.83, 1.15)
Tertile 2 vs. Tertile 1	0.94 (0.63, 1.40)	0.86 (0.59, 1.26)
Tertile 3 vs. Tertile 1	1.15 (0.78, 1.70)	0.92 (0.63, 1.35)
450-CpG score		
Continuous score	1.19 (1.00, 1.41)	0.98 (0.85, 1.14)
Tertile 2 vs. Tertile 1	0.83 (0.55, 1.25)	0.96 (0.65, 1.41)
Tertile 3 vs. Tertile 1	1.44 (1.00, 2.09)	1.10 (0.75, 1.60)
**CRC-related survival**		
3-CpG score		
Continuous score	1.18 (1.00, 1.39)	**1.18 (1.02, 1.36)**
Tertile 2 vs. Tertile 1	1.06 (0.74, 1.51)	**1.17 (0.81, 1.69)**
Tertile 3 vs. Tertile 1	1.19 (0.83, 1.72)	**1.45 (1.01, 2.07)**
144-CpG score		
Continuous score	0.89 (0.75, 1.07)	1.07 (0.92, 1.24)
Tertile 2 vs. Tertile 1	0.91 (0.65, 1.27)	0.94 (0.66, 1.34)
Tertile 3 vs. Tertile 1	0.82 (0.58, 1.18)	1.19 (0.84, 1.69)
450-CpG score		
Continuous score	0.98 (0.84, 1.15)	0.97 (0.85, 1.12)
Tertile 2 vs. Tertile 1	1.46 (1.04, 2.05)	0.93 (0.66, 1.32)
Tertile 3 vs. Tertile 1	1.04 (0.72, 1.52)	0.89 (0.62, 1.27)

eTable 6. Associations between methylation-based alcohol consumption scores and mortality by sex among patients with stage I-III CRC

	Blood sample, HR (95% CI)		Tumor tissue sample, HR (95% CI)	
	**Male (*N* = 1068)**	**Female (*N* = 766)**	**Male (*N* = 1325)**	**Female (*N* = 945)**
**Overall survival**				
3-CpG score				
Continuous score	1.15 (1.06, 1.23)	1.28 (1.14, 1.43)	1.04 (0.95, 1.14)	1.01 (0.91, 1.12)
Tertile 2 vs. Tertile 1	1.02 (0.82, 1.27)	0.93 (0.71, 1.23)	1.02 (0.83, 1.26)	1.08 (0.83, 1.40)
Tertile 3 vs. Tertile 1	1.34 (1.09, 1.66)	1.35 (1.03, 1.76)	1.10 (0.89, 1.35)	1.02 (0.78, 1.33)
144-CpG score				
Continuous score	1.04 (0.96, 1.14)	0.97 (0.85, 1.11)	0.97 (0.89, 1.06)	1.06 (0.95, 1.18)
Tertile 2 vs. Tertile 1	0.99 (0.80, 1.21)	0.84 (0.65, 1.09)	0.91 (0.75, 1.11)	0.90 (0.69, 1.16)
Tertile 3 vs. Tertile 1	1.03 (0.84, 1.28)	0.93 (0.72, 1.21)	0.88 (0.72, 1.08)	1.07 (0.83, 1.39)
450-CpG score				
Continuous score	1.10 (1.01, 1.20)	1.03 (0.92, 1.16)	0.93 (0.86, 1.01)	1.09 (0.98, 1.22)
Tertile 2 vs. Tertile 1	1.16 (0.94, 1.43)	0.90 (0.70, 1.16)	0.99 (0.81, 1.21)	0.90 (0.70, 1.17)
Tertile 3 vs. Tertile 1	1.16 (0.94, 1.44)	0.92 (0.70, 1.20)	0.96 (0.78, 1.17)	1.22 (0.95, 1.57)
**Non-CRC-related survival**				
3-CpG score				
Continuous score	1.19 (1.09, 1.31)	1.34 (1.15, 1.56)	1.07 (0.95, 1.20)	0.94 (0.81, 1.08)
Tertile 2 vs. Tertile 1	1.05 (0.79, 1.39)	0.98 (0.66, 1.45)	0.92 (0.70, 1.20)	0.98 (0.70, 1.38)
Tertile 3 vs. Tertile 1	1.49 (1.13, 1.95)	1.50 (1.04, 2.18)	1.08 (0.83, 1.40)	0.85 (0.59, 1.21)
144-CpG score				
Continuous score	1.07 (0.96, 1.19)	1.11 (0.92, 1.35)	0.95 (0.85, 1.06)	1.02 (0.89, 1.18)
Tertile 2 vs. Tertile 1	0.94 (0.72, 1.23)	1.16 (0.80, 1.66)	0.91 (0.71, 1.17)	0.94 (0.67, 1.34)
Tertile 3 vs. Tertile 1	1.08 (0.83, 1.40)	1.29 (0.88, 1.87)	0.86 (0.66, 1.12)	0.98 (0.69, 1.39)
450-CpG score				
Continuous score	1.17 (1.05, 1.30)	1.12 (0.99, 1.27)	0.90 (0.81, 1.00)	1.16 (1.00, 1.34)
Tertile 2 vs. Tertile 1	1.16 (0.88, 1.53)	1.02 (0.73, 1.44)	0.99 (0.77, 1.27)	1.00 (0.70, 1.44)
Tertile 3 vs. Tertile 1	1.42 (1.08, 1.85)	1.06 (0.73, 1.52)	0.90 (0.69, 1.18)	1.59 (1.13, 2.23)
**CRC-related survival**				
3-CpG score				
Continuous score	1.07 (0.94, 1.23)	1.18 (0.99, 1.41)	1.01 (0.87, 1.17)	1.10 (0.94, 1.29)
Tertile 2 vs. Tertile 1	0.97 (0.69, 1.38)	0.83 (0.55, 1.25)	1.22 (0.87, 1.71)	1.25 (0.83, 1.90)
Tertile 3 vs. Tertile 1	1.10 (0.76, 1.58)	1.14 (0.77, 1.71)	1.14 (0.80, 1.61)	1.34 (0.89, 2.04)
144-CpG score				
Continuous score	0.99 (0.85, 1.15)	0.81 (0.67, 0.99)	1.03 (0.89, 1.18)	1.06 (0.89, 1.25)
Tertile 2 vs. Tertile 1	1.06 (0.75, 1.48)	0.52 (0.35, 0.77)	0.94 (0.67, 1.32)	0.77 (0.51, 1.17)
Tertile 3 vs. Tertile 1	0.92 (0.63, 1.33)	0.61 (0.41, 0.91)	0.96 (0.68, 1.36)	1.08 (0.73, 1.59)
450-CpG score				
Continuous score	0.99 (0.86, 1.15)	0.90 (0.74, 1.10)	1.02 (0.89, 1.17)	1.00 (0.85, 1.19)
Tertile 2 vs. Tertile 1	1.25 (0.90, 1.75)	0.79 (0.54, 1.16)	1.02 (0.72, 1.45)	0.83 (0.56, 1.24)
Tertile 3 vs. Tertile 1	0.80 (0.54, 1.16)	0.80 (0.53, 1.20)	1.14 (0.81, 1.59)	0.85 (0.57, 1.27)

aHR = adjusted hazards ratio; CI = confidence interval.

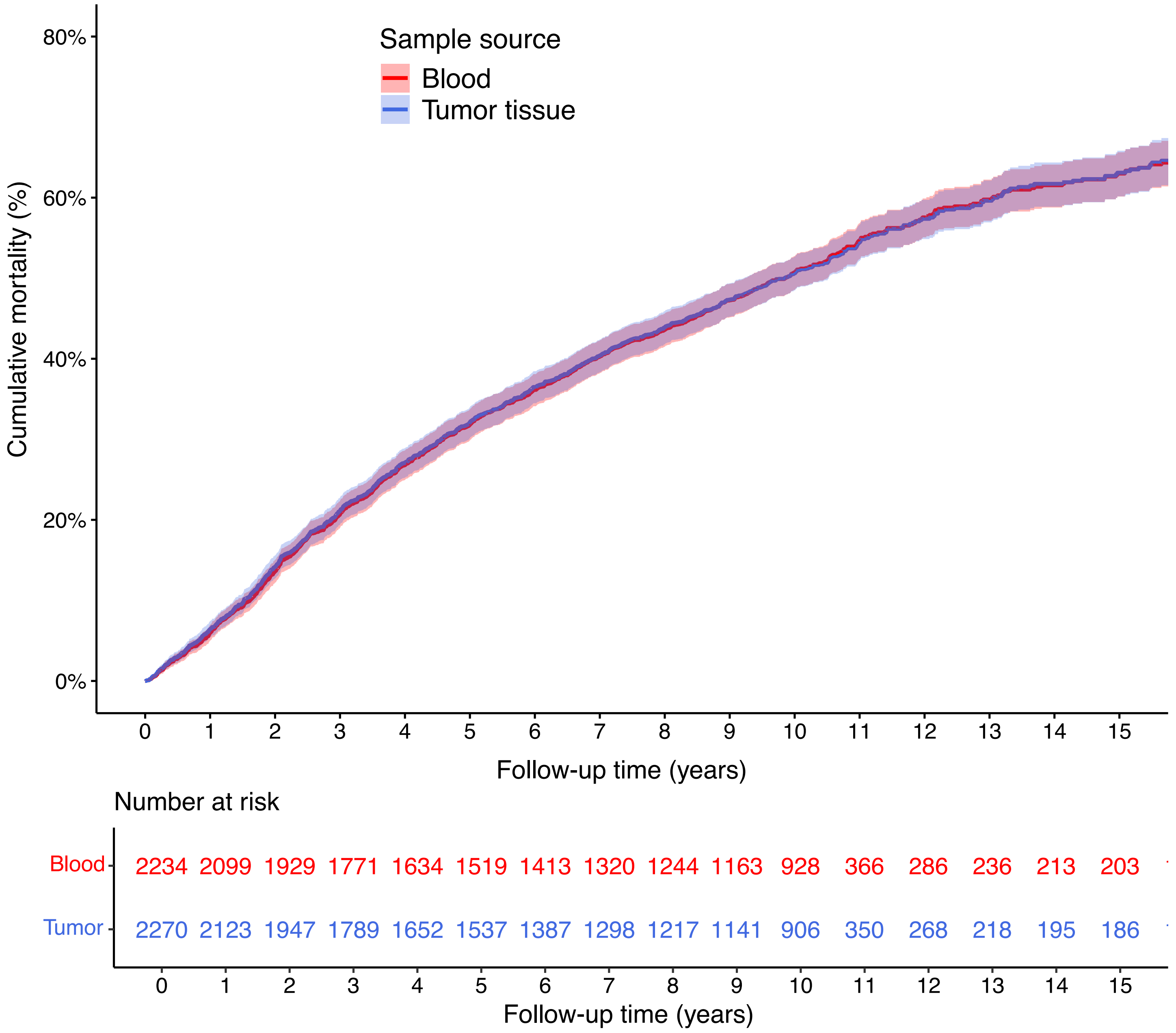

eFigure 1 Cumulative mortality curves comparing patients with blood methylation data and those with tumor methylation data

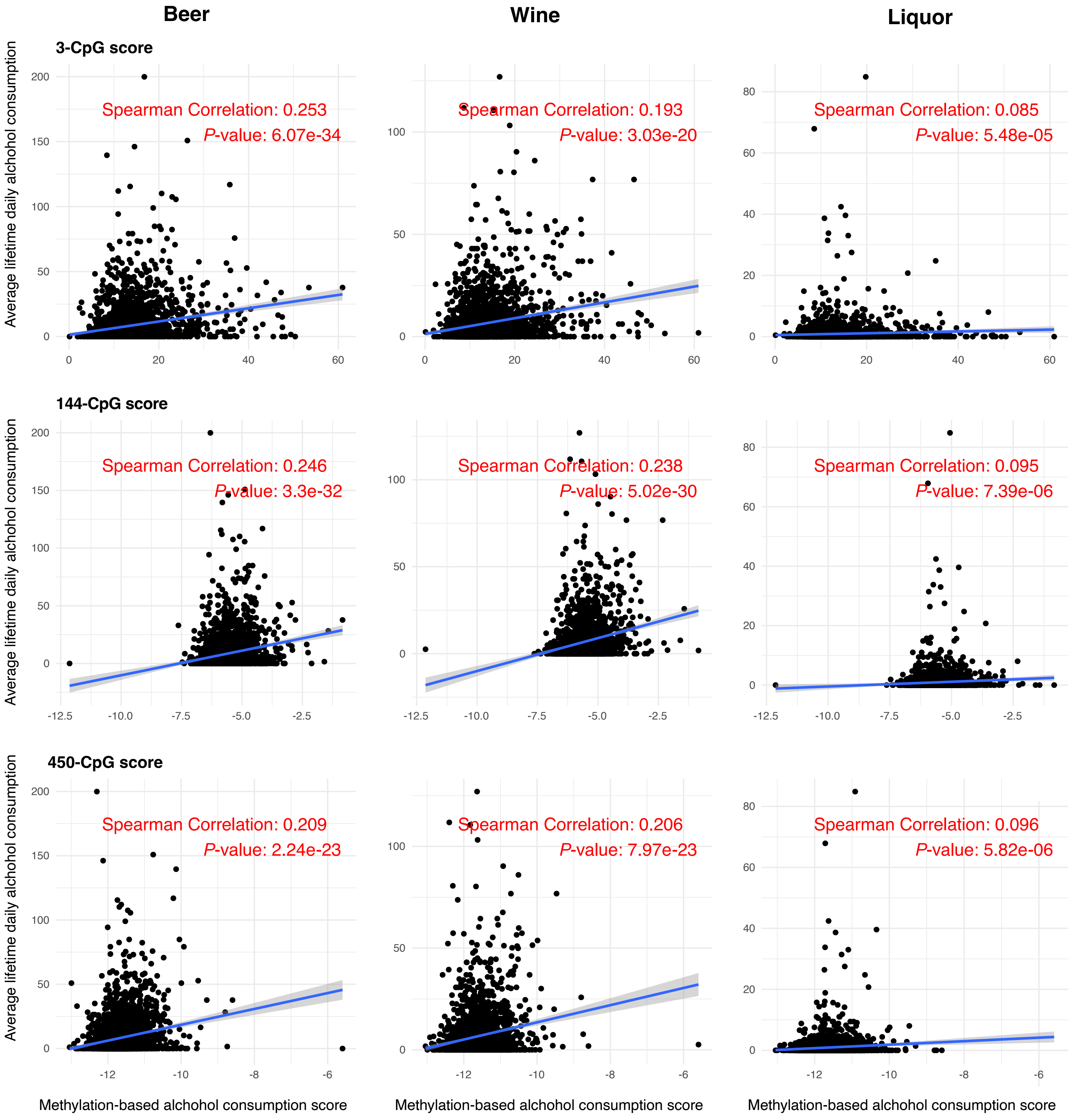

eFigure 2 The associations between methylation-based alcohol consumption score and self-reported average lifetime daily beverage-specific alcohol intake

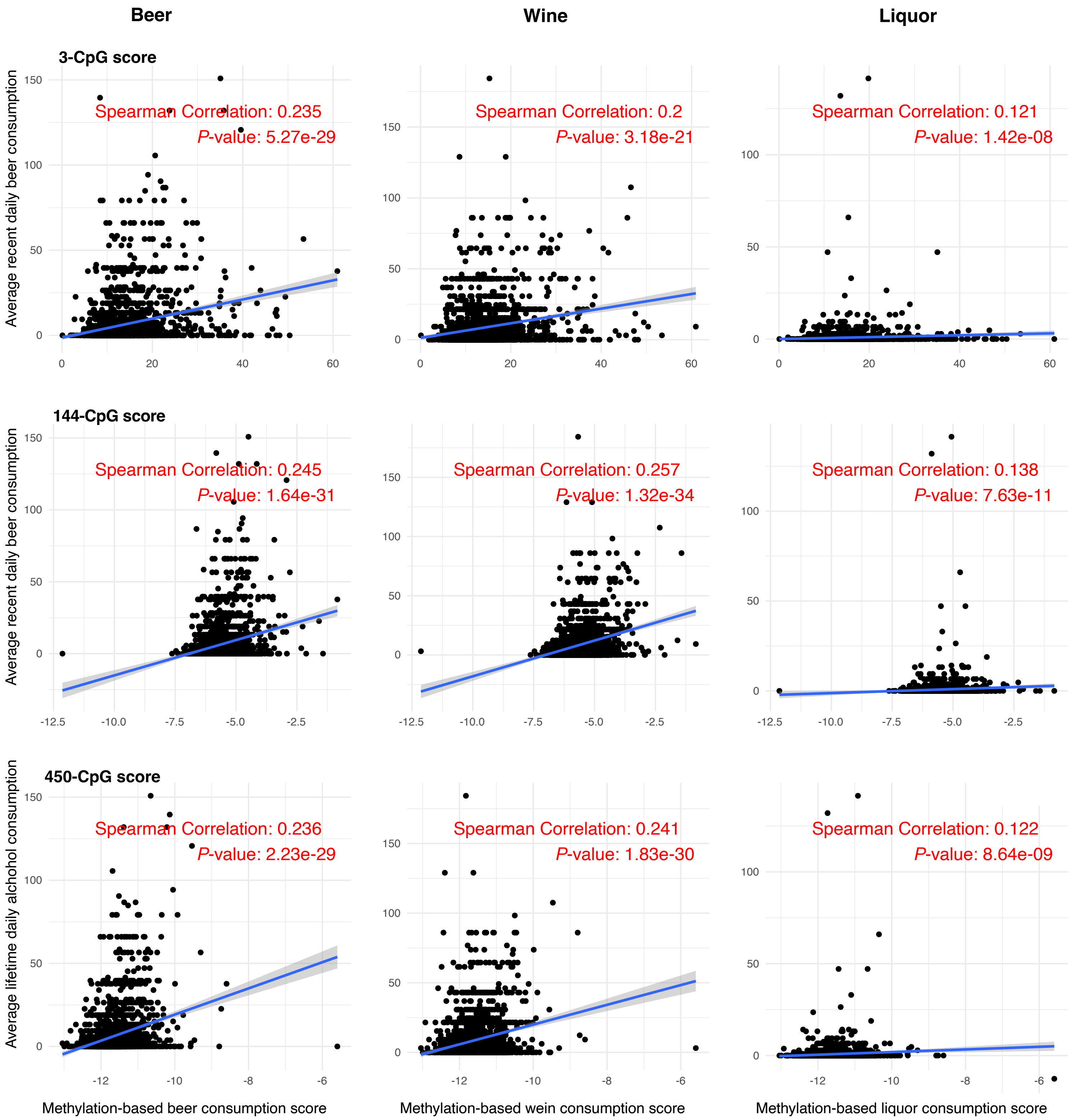

eFigure 3 The association between methylation-based alcohol consumption score and self-reported average recent daily beverage-specific alcohol intake

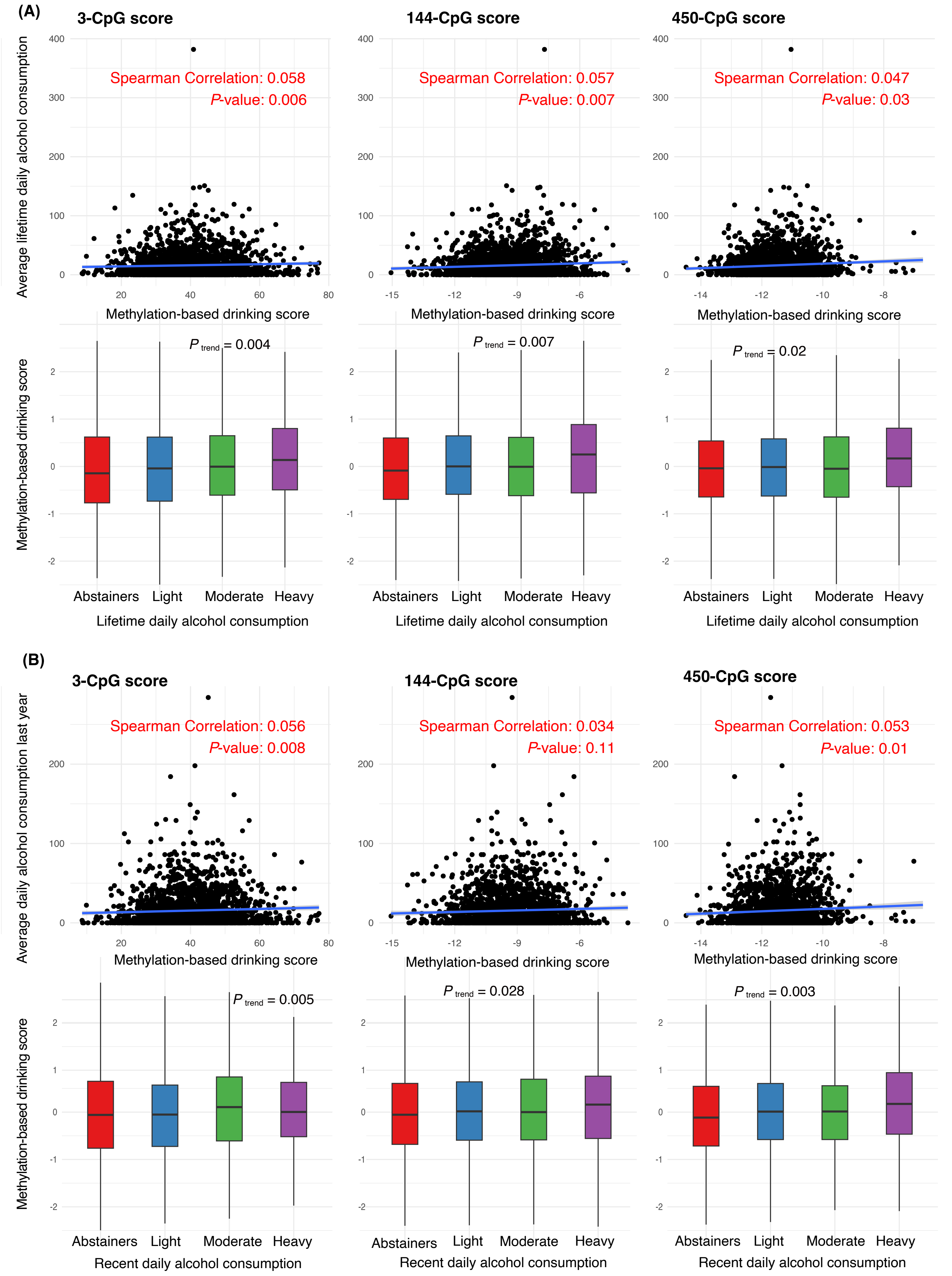

Figure 4. Associations between tumor methylation-based alcohol consumption scores self-reported daily alcohol consumption levels. (A) Lifetime alcohol consumption; (B) Recent alcohol consumption

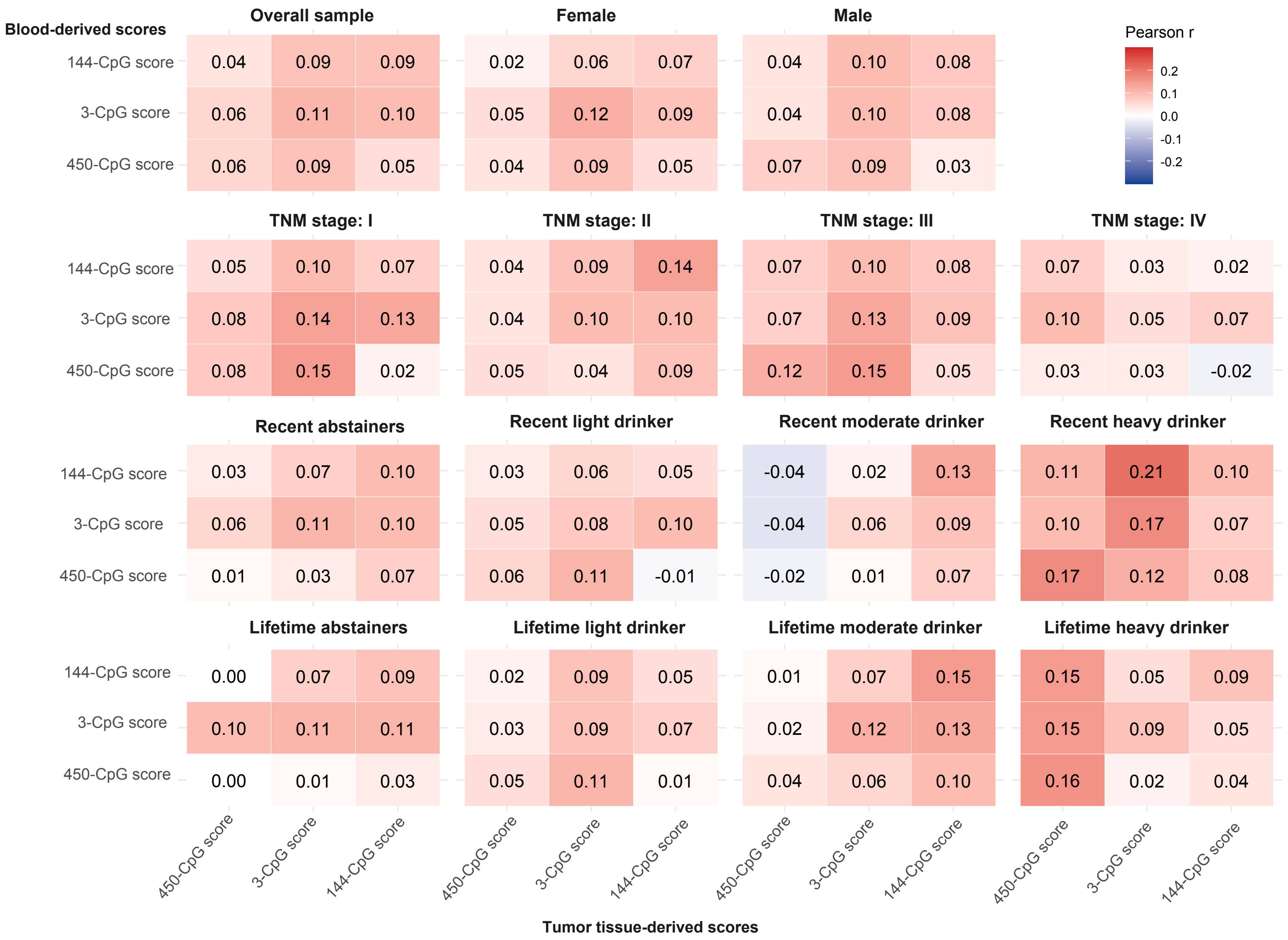

Figure 5. Pearson correlation r values between tumor- and blood-derived methylation scores among overlapping patients (n = 1906) overall, and by sex, TNM stage, and self-reported drinking behaviors.
